# Number of seasonal exposures to Japanese cedar pollen increases the risk of sensitization: Observational study in Korean adults

**DOI:** 10.1038/s41598-019-47124-5

**Published:** 2019-07-19

**Authors:** Michelle J. Suh, Hee Jun Yi, Jeong Hong Kim, Keun-Hwa Lee, Sung-Chul Hong, Ju wan Kang

**Affiliations:** 1 Department of Otorhinolaryngology, Jeju National University College of Medicine, Jeju, Republic of Korea; 2 The Environmental Health Center (Atopic Dermatitis and Allergic Rhinitis), Jeju National University, Jeju, Republic of Korea

**Keywords:** Epidemiology, Risk factors

## Abstract

Sensitization to seasonal allergens usually requires repeated exposure to them. However, research on the extent of exposure that increases the risk of sensitization to specific allergens is lacking. Therefore, we investigated the levels of exposure to Japanese cedar pollen that increased the risk of sensitization to it. A cross-sectional study was conducted with 857 college students living in Jeju, South Korea, as it is the only province in Korea where Japanese cedar pollen levels are high. Questionnaires about demographic characteristics were distributed and skin prick tests for allergic sensitization were performed. Sensitization rates of groups divided by residence period were 3.8% (less than 1 year), 1.8% (1–2 years), 8.5% (2–3 years), 10.3% (3–4 years), 14.8% (4–10 years), and 19.1% (over 10 years). Residence period was an influencing factor of sensitization rate to Japanese cedar pollen, and the cut-off value of the residence period that increased the risk of sensitization to Japanese cedar pollen was found to be 25 months. Repeated exposure to seasonal allergens was related to an increased sensitization rate in young adults. Our results suggested that exposure to Japanese cedar pollen for over two seasons could increase the risk in Korean adults.

## Introduction

Allergic rhinitis (AR) is an IgE-mediated inflammatory disease that occurs after exposure of the nasal mucosa to allergens, and is characterized by clear nasal discharge, nasal congestion, and sneezing^1,2^. Allergic diseases are increasing worldwide^3–5^ leading to a decrease in quality of life and an increase in medical costs. Hence, the need for precise data on diseases and risk factors has increased worldwide for efficient management of diseases^6^. It is difficult to study the effect of individual factors because the prevalence and course of allergic diseases differ due to genetic and environmental factors being involved in their onset and progression^7^. Environmental factors are the major known risk factors for AR. Sensitization is the first step in the immunological response to allergens, and the allergen-specific IgE antibodies formed by sensitization lead to allergic rhinitis on re-exposure to the allergens^8^. In general, repeated exposure to an antigen is known to increase susceptibility to sensitization by an antigen and the association between allergen exposure and allergic sensitization might provide important information of progression of allergic disease^9^. However, there has been a lack of studies on the direct association between allergic sensitization and features of allergen exposure (such as dose or period).

Jeju island, the southernmost and geographically confined island off the Korean peninsula, has the largest distribution of Japanese cedar (JC, *Cryptomeria japonica*) trees in the country^10,11^. It is the most frequent outdoor sensitizer in Jeju and the rate of sensitization by JC pollen in this area is as high as 33.8%, whereas in Seoul it is as low as 1.1%^12–14^. With the geographically specific allergen, we investigated whether an association between the duration of seasonal exposure to Japanese cedar pollen and allergic sensitization exists and whether the length of exposure to JC pollen increases this risk.

## Results

### Population characteristics by residential period in Jeju and Prevalence of sensitization

A total of 857 participants were enrolled in this cross-sectional study (Table 1). The group consisted of 369 (43.1%) males, aged 19–34 years, with a mean of 22.92 ± 2.856 (standard deviation) years. The mean duration of their stay in Jeju island was 14.6 ± 9.8 years [range, 0.8–34]. When the participants were grouped by residency period, 64.3% of the responders had been residents for more than 10 years, followed by residents for less than one year (15.5%). Parental allergic disease was defined as, “at least one of the parents diagnosed with asthma, atopic dermatitis (AD) or AR”, which accounted for 32.1% of the total subjects. Further, 11.8% were smokers, 41.9% had a history of diagnosed rhinitis, and 7.5% were diagnosed with asthma.

**Table 1:** Subject characteristics and demographics.

Variables	Study population (n = 857)
Sex (Male:Female)	369 (43.1%):488 (56.9%)
Age (year) [Range]	22.92 ± 2.856 [19–34]
Residence duration (year) [Range]	14.6 ± 9.8 [0.8–34]
≤12 months	131 (15.5%)
12–24 months	56 (6.6%)
24–36 months	59 (7.0%)
36–48 months	29 (3.4%)
48–120 months	27 (3.2%)
>120 months	544 (64.3%)
BMI	22.51 ± 3.35 [15.06–38.90]
Smoking	101 (11.8%)
Parental Allergic disease Hx	265 (32.1%)
Rhinitis Dx	346 (41.9%)
Asthma Dx	64 (7.5%)

Mean SD was reported for age.

Abbreviation: Hx: history, Dx: diagnosis.

**Figure 1:**
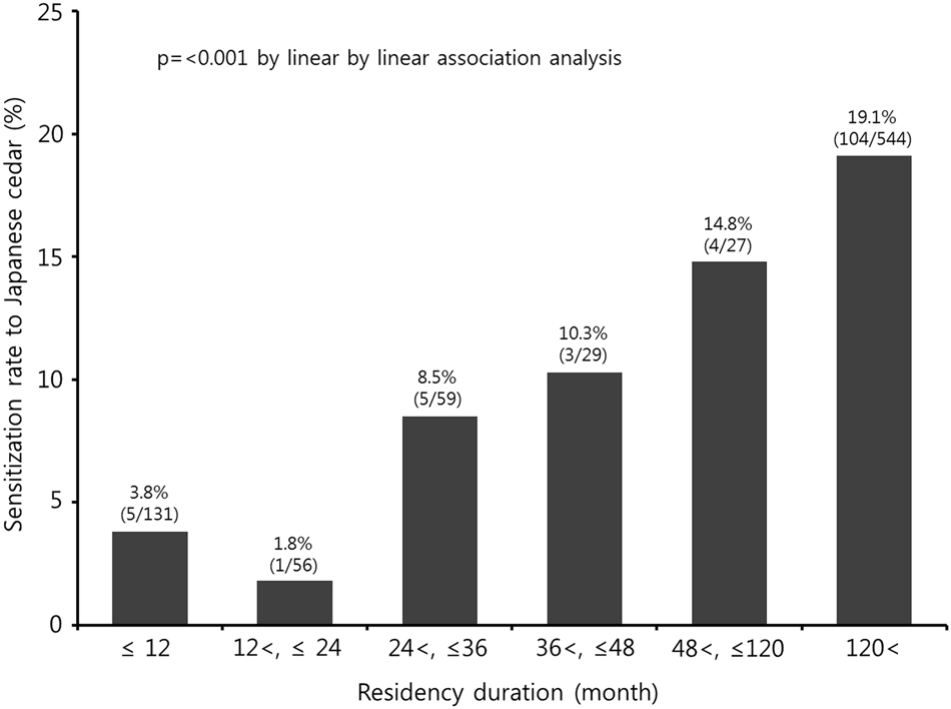
Specific allergen sensitization rate according to duration of residence. *P*-values were calculated using an analysis of variance.

The overall prevalence rate of a positive reaction to Japanese Cedar pollen extract in the skin prick test was 14.4%. JC sensitization rate of each group divided by residency period was 3.8% (≤12 months), 1.8% (12–24 months), 8.5% (24–36 months), 10.3% (36–48 months), 14.8% (48–120 months), and 19.1% (>120 months), respectively. In the linear-by-linear analysis, the sensitivities of the six groups according to the residence time were found to increase with the duration of residence (p < 0.001; Fig. 1).

### Univariate analysis

Univariate logistic regression analysis was performed to evaluate the risk factors for JC sensitization, including confounding variables such as parental AR history, body mass index (BMI), smoking, age, sex, history of rhinitis diagnosis, asthma, and duration of stay as independent variables and JC sensitization as a dependent variable (Table 2). Statistically significant risk factors were parental allergic disease history, residency duration, history of rhinitis, and asthma diagnosis. Higher age and smoking increased the odds of sensitization by 1.085 [95% confidence interval (CI) = 0.363–3.245] and although not statistically significant, was lower in men than in women.

**Table 2:** Univariate logistic regression analysis for identifying the risk factors for JC sensitization.

	Odds ratio	95% CI	P value
Parental Allergic disease	1.526	1.125 to 2.070	0.007^†^
BMI	0.992	0.935 to 1.053	0.799
Smoking	1.270	0.672 to 2.400	0.461
Residential period (months)	1.005	1.003 to 1.008	<0.001^†^
Age	1.065	0.926 to 1.225	0.375
Male versus female	0.858	0.329 to 2.238	0.755
History of rhinitis Dx	2.840	1.896 to 4.254	<0.001^†^
Asthma Dx	1.948	1.054 to 3.598	0.033^†^

^†^Significant difference (p < 0.05) between groups; BMI, body mass index; CI, confidence interval.

Abbreviation: Dx: diagnosis.

### Multivariate analysis

Multivariate logistic regression analysis was performed using independent variables that showed significance in the univariate logistic regression analysis and JP sensitization as the dependent variable. When analysis was performed for all subjects, the presence of asthma diagnosis [odds ratio (OR), 5.276; CI, 1.447–19.238; p = 0.012] and residential period (OR, 1.021; CI, 1.003–1.039; p = 0.025) were found to be significant risk factors of JC sensitization (Table 3). Moreover, the multivariate logistic regression analysis for the subjects with a residence period less than 10 years also showed that history of asthma diagnosis (OR, 5.276; CI, 1.447–19.238; p = 0.01) and the residence period (OR, 1.020; CI, 1.002–1.038; p = 0.028) were independent risk factors for allergic sensitization to JC (Table 4). In both analyses, neither the history of diagnosed rhinitis nor parental allergic disease were statistically significant.

**Table 3:** Multiple logistic regression analysis for identifying the risk factors for JC sensitization in overall subjects.

	P value	Odds ratio	95% CI
Asthma Dx	0.012^†^	5.276	1.447 to 19.238
History of rhinitis Dx	0.091	2.876	0.846 to 9.781
Residential period (months)	0.025^†^	1.021	1.003 to 1.039
Parental allergic disease	0.27	1.656	0.676 to 4.059

**Table 4:** Multiple logistic regression analysis for identifying the risk factors for JC sensitization in dwellers under 10 years (n = 309).

	P value	Odds ratio	95% CI
Asthma Dx	0.010^†^	5.472	1.512 to 19.808
History of rhinitis Dx	0.053	3.600	0.982 to 13.196
Residential period (months)	0.028^†^	1.020	1.002 to 1.038
Parental allergic disease	0.537	1.428	0.461 to 4.425

^†^Significant difference (p < 0.05) between groups.

Abbreviation: Dx: diagnosis.

### Analysis of ROC curves for sensitivities to residency periods

Based on the assumption that residents were migrants who stayed in the area for less than 10 years, ROC curve analysis was performed for ‘residency period’ which showed significant correlation in multivariate analysis. We could obtain 25 months as the cut-off value for residency period associated with increasing risk of JC sensitization. When the residency period was divided into two groups (A: <25 months, B: ≥25 months but <120 months) using 25 months as the cutoff value, JC sensitization rate was significantly higher in subjects who were residents for ≥25 months but <120 months (10.4%) compared to subjects who were residents for less than 25 months (3.2%) (OR, 3.515; CI, 1.281–9.644; p value = 0.01).

## Discussion

The purpose of this study was to elucidate the correlation between the duration of seasonal allergen exposure and sensitization using a region-specific allergen in Jeju. Jeju Island has the largest distribution of Japanese cedar trees in South Korea and their pollen is in abundance from late January to mid-April, and especially early March. JC pollen from Jeju has the highest sensitization rate in Korea. The sensitization rates in subjects with residency periods of less than two years (3.2%), which were somewhat higher than those from other regions as reported in previous studies, showed the lowest prevalence in overall dwellers^12–14^. Further, it increased as the residence period increased, rising to 19.1% when the residence period reached 10 years and over. However, sensitization rates do not seem to increase any further after 120 months of exposure to Cedar pollen. Furthermore, this might indicate that the sensitization rates reach a plateau after the passage of a certain duration. However, other allergens including seasonal and perennial allergens did not show any significant change along with residency duration (data not shown).

We could conclude that longer duration of stay in Jeju province was associated with increased odds of JC sensitization in young adults. In addition, 25 months of residence in Jeju Island area was a diagnostic cutoff period of sensitization. Residents within the cutoff period and over had odds ratios of JC sensitization that were 3.515 times higher (p = 0.01) than that of residents with a residency of under 25 months. Asthma history was associated with increased risk of JC sensitization, which was compatible with previous reports^11,12^. However, other factors including parental allergy history and history of diagnosed rhinitis did not show a significant association with JC sensitization.

Previous studies focused on sensitization and the timing of lifetime allergen exposure and their effect on disease progression^15,16^ have concluded that exposure in early childhood, especially in infancy, was an important factor for sensitization and had a risk of developing into allergic diseases. High levels of birch pollen exposure in infancy increased the risk of sensitization to the same allergen as well as the risk of allergic asthma^17^. Additional experimental evidence supports the importance of primary sensitization to inhalant allergens in AR in early infancy. The transient deficiency of IgA at this stage facilitates the permeation of the allergen through the mucosa of intestinal and bronchial walls. This period is the determining factor whether the sensitization will lead to allergic reactivity in adulthood^18,19^.

According to previous studies, allergic sensitization increases with repeated exposure to various outdoor antigens after a critical period when a person’s immunological maturation is complete. Two studies of adult immigrants in the US have confirmed an increase in prevalence of allergic respiratory diseases according to the duration of residence, regardless of history of origin or age, indicating that exposure to respiratory triggers in household environment or workplaces influences respiratory health^20,21^. However, the study did not examine the association between types of aeroallergens (perennial, seasonal, or occupational) and diseases. Even with all of these, the relationship between the load (duration and amount) of allergen exposure and sensitization could not be clearly defined. Moreover, the relationship between a specific allergen and exposure duration is difficult to identify^22^. Several studies on exposure to allergens have shown that sensitization tends to increase if the concentration of allergens goes up to plateau, but on the contrary, if the concentration is too high, the sensitization rate is decreased (TH2 priming is preferentially favored by low-dose antigen exposure, whereas higher doses favor TH1 priming)^23–25^. According to their hypotheses, allergen load affects sensitization according to the baseline exposure in the target environment. It is necessary to further investigate whether this effect is equally applicable to the relationship between sensitization and duration of seasonal allergen exposure.

To our knowledge, ours is the first study that uniquely identifies the relationship between duration of allergen exposure and sensitization resulting from long-term exposure to allergen that increases sensitization rate after early childhood.

We concluded that sensitization in a new environment generally increases after a certain period of time in immunologically mature subjects. Differences may exist in adults, but accurate and rapid detection at appropriate times is considered to be able to modify disease progression. A residency duration of over 25 months can be considered as a risk factor as well as a proper time to evaluate for newly developed JC pollen sensitization, because our results showed that sensitization to JC pollen is determined by average residency duration with at least two seasonal exposures.

The strength of our research is that it provides information on the response of adults exposed to a new allergen. However, as the study was only a yearlong cross-sectional study, there were limitations in clarifying the time-related causal relationship between allergic diseases and risk factors, hence care needs to be exercised in interpreting the results. First, apart from the period of residence, some of the subjects may have had a history of exposure to JC which was not excluded. Further, since various cypress pollen including Cryptomeria allergens, Thuja, and Chamaecyparis strongly cross-react with JC pollen^26,27^, an evaluation of exposure of these cypress pollen to confirm the exact causal relationship between sensitization and JC pollen exposure according to residency duration is needed. Also, inclusion of skin prick test results of other cypress pollen (Cupressus, Juniperus, etc.) for analysis might yield a better result. Second, we performed a skin prick test but no serologic test for specific IgE. As mentioned before, since JC pollen could show cross-reactivity with other cypress pollen, data about IgE antibodies against the major Cryptomeria allergen Cry j 1 will be valuable in excluding any cross-reactivity in skin prick test. Third, we did not consider the subjects’ symptoms. Allergic sensitization does not necessarily indicate clinical allergy, hence, the subjects’ symptoms should be considered for clinical significance. Moreover, the odds ratio of JC pollen sensitization related to residency duration calculated in this study is significant but very low, thus prompting the consideration of the clinical significance of this result. Finally, we studied JC pollen exposure with regard to residency duration only. However, JC pollen load, intensity during each season, and daily outdoor exposure might affect the risk of sensitization. A research study in preschool children showed longer duration of outdoor activities increased the odds of epidemiological AR but not clinical AR^28^. Therefore, these factors need to be considered for concrete conclusions.

## Conclusions

Longer exposure to region-dominant seasonal allergens increases sensitization rates. We postulated that two seasonal exposures might increase the risk of sensitization to Japanese cedar pollen in Korean adults. A longitudinal study commencing from the time of migration of the subjects relating to the onset and progression of AR with every passing season needs to be conducted.

## Materials and Methods

### Study design and participants

Data from students studying in Jeju National University, who were enrolled in this study, was collected in September 2016. Detailed information about the study (skin prick test and questionnaire) was given to the subjects, and informed consent was obtained from all enrolled subjects. The questionnaires were distributed and returned. Further, skin prick tests were performed. The subjects who did not return the questionnaires and/or undergo the skin prick test were excluded from the analysis. The Institutional Review Board of Jeju National University Hospital approved this study in accordance with the Declaration of Helsinki.

### Assessment

#### Questionnaires

Questionnaires consisted of demographic characteristics (age, sex, and college grade), and asthma or rhinitis diagnosis, presence of nasal allergic symptoms, parental allergic rhinitis, economic level, health status, smoking status, BMI, and residence period in Jeju.

#### Skin prick test (SPT)

In addition to Japanese cedar tree pollen, 24 aeroallergens purchased from Allergopharma (Reinbek, Germany) were tested for sensitization. *Pinus densiflora*, (pine), *Dermatophagoides pteronyssinus* (dust mite), *Salix koreensis*, (willow), *Acer palmatum*, (maple), *Betula platyphylla*, (birch), *Quercus* (oak), *Ambrosia artemisiifolia* (ragweed), *Penicillium*, *Cynodon dactylon* (bermuda grass), *Artemisia princeps* (mugwort), *Alnus japonica* (alder), *Phleum pratense* (timothy grass), *Aspergillus*, *Cladosporium, Alternaria*, *Humulus japonicus* (Japanese hop), *Lolium perenne* (ryegrass), *Dactylis glomerata* (orchard grass), *Festuca ovina* (sheep fescue), *Ulmus davidiana* (elm), *Anthoxanthum odoratum* (vernal grass), *Chenopodium album* (fat hen), *Plantago asiatica* (plantain) were evaluated. For Japanese cedar, a commercial allergen was purchased (Greer Laboratories Inc., Lenoir, NC, USA). Histamine hydrochloride at a concentration of 1 mg/mL (Allergopharma, Reinbek, Germany) and normal saline solution with 50% glycerin were used as positive and negative controls, respectively. SPT was performed on the forearms by trained researchers using a 23-G lancet. Fifteen minutes after SPT, the size of each wheal was measured as the mean of (A) the longest diameter and (B) the diameter perpendicular to it at the midpoint [i.e., (A + B)/2]. A positive skin reaction to an allergen was defined if the allergen elicited a wheal ≥3 mm. Allergic sensitization was established when the subject showed a positive skin test to each allergen. Prior to testing, all eligible participants were instructed to stop taking all medicines containing antihistamine for at least 72 h before the examination.

### Statistical analysis

The descriptive statistics were expressed as percentage (%) or mean ± standard deviation (mean ± SD). Linear-by-linear association analysis was used to analyze the proportion of sensitization or presence of symptoms between age groups. A useful subset of predictors among many variables was identified using stepwise multiple regression analysis. Receiver operation curve analysis was performed to determine the cut-off value. Data were analyzed using SPSS (ver18.0 for Windows, SPSS Inc, Chicago, IL, USA) A p-value < 0.05 (two-tailed) was considered statistically significant.

## Data Availability

All available data generated or analyzed during this study are included in this published article. Other raw data are not available because of regulation of data sharing in the Republic of Korea.
